# Temporal scale of habitat selection for large carnivores: Balancing energetics, risk and finding prey

**DOI:** 10.1111/1365-2656.13613

**Published:** 2021-10-29

**Authors:** Anna C. Nisi, Justin P. Suraci, Nathan Ranc, Laurence G. Frank, Alayne Oriol‐Cotterill, Steven Ekwanga, Terrie M. Williams, Christopher C. Wilmers

**Affiliations:** ^1^ Center for Integrated Spatial Research Environmental Studies Department University of California Santa Cruz CA USA; ^2^ Conservation Science Partners Truckee CA USA; ^3^ Living with Lions Mpala Research Centre Nanyuki Kenya; ^4^ Museum of Vertebrate Zoology University of California Berkeley CA USA; ^5^ Wildlife Conservation Research Unit Zoology Department Oxford University Abingdon UK; ^6^ Lion Landscapes Teignmouth UK; ^7^ Ecology and Evolutionary Biology Department University of California Santa Cruz CA USA

**Keywords:** habitat selection, human‐dominated landscapes, integrated step selection analysis, *Panthera leo*, *Puma concolor*, spatio‐temporal scale, temporal grain

## Abstract

1. When navigating heterogeneous landscapes, large carnivores must balance trade‐offs between multiple goals, including minimizing energetic expenditure, maintaining access to hunting opportunities and avoiding potential risk from humans. The relative importance of these goals in driving carnivore movement likely changes across temporal scales, but our understanding of these dynamics remains limited.
2. Here we quantified how drivers of movement and habitat selection changed with temporal grain for two large carnivore species living in human‐dominated landscapes, providing insights into commonalities in carnivore movement strategies across regions.
3. We used high‐resolution GPS collar data and integrated step selection analyses to model movement and habitat selection for African lions *Panthera leo* in Laikipia, Kenya and pumas *Puma concolor* in the Santa Cruz Mountains of California across eight temporal grains, ranging from 5 min to 12 hr. Analyses considered landscape covariates that are related to energetics, resource acquisition and anthropogenic risk.
4. For both species, topographic slope, which strongly influences energetic expenditure, drove habitat selection and movement patterns over fine temporal grains but was less important at longer temporal grains. In contrast, avoiding anthropogenic risk during the day, when risk was highest, was consistently important across grains, but the degree to which carnivores relaxed this avoidance at night was strongest for longer term movements. Lions and pumas modified their movement behaviour differently in response to anthropogenic features: lions sped up while near humans at fine temporal grains, while pumas slowed down in more developed areas at coarse temporal grains. Finally, pumas experienced a trade‐off between energetically efficient movement and avoiding anthropogenic risk.
5. Temporal grain is an important methodological consideration in habitat selection analyses, as drivers of both movement and habitat selection changed across temporal grain. Additionally, grain‐dependent patterns can reflect meaningful behavioural processes, including how fitness‐relevant goals influence behaviour over different periods of time. In applying multi‐scale analysis to fine‐resolution data, we showed that two large carnivore species in very different human‐dominated landscapes balanced competing energetic and safety demands in largely similar ways. These commonalities suggest general strategies of landscape use across large carnivore species.

## Graphical abstract

During movement, large carnivores must both minimize energetic expenditure and avoid anthropogenic risk. The authors found these goals influence fine‐scale and longer term movements differently, and identify patterns between two different systems, lions in a rangeland system and pumas in an urban‐adjacent area, suggesting commonalities in carnivore movement strategies in human‐dominated landscapes.

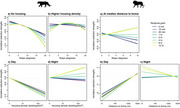

## 1 INTRODUCTION

Habitat selection, defined as disproportionate use of habitat features relative to their availability, provides a window into the drivers of animal decision‐making by reflecting how animals balance fitness‐related goals as they move around a landscape (McLoughlin et al., 2006; Rosenzweig, 1981). Habitat selection has long been recognized as a scale‐dependent process, and often, drivers of habitat selection change depending on the scale of analysis (Boyce, 2006; Johnson, 1980; Mayor et al., 2009; McGarigal et al., 2016). Comparing habitat selection across spatio‐temporal scales (i.e. longer term over larger distances versus shorter term over smaller distances) can reveal fitness‐relevant trade‐offs and hierarchical relationships between goals that would not be apparent if only a single scale was considered, which can have implications for conservation and management (Bastille‐Rousseau et al., 2015; Hebblewhite & Merrill, 2009; Rettie & Messier, 2000).

The concept of scale encompasses both grain (the spatial or temporal resolution of data; e.g. pixel size for spatial covariates or how frequently animal locations are sampled) and extent (size of study area in space and/or duration of study in time; McGarigal et al., 2016; Wheatley & Johnson, 2009). Much scale‐dependent habitat selection research has focused either on the spatial grain of habitat covariates or on comparing selection across broad spatio‐temporal scales (McGarigal et al., 2016), which can elucidate drivers of behaviours that operate over longer periods of time (e.g. days to weeks to months) including migration, dispersal and territoriality (Bastille‐Rousseau et al., 2015; DeCesare et al., 2012; Hebblewhite & Merrill, 2009; Zeller et al., 2017). The relative importance of landscape features also likely varies over finer temporal grains (i.e. within a day), and broader cross‐scale comparisons may overlook important drivers and trade‐offs of short‐term habitat selection. For example, short‐term movement over the course of minutes may be driven by fine‐grain topographic variation that determines how much energy an animal must expend during each movement event (Nickel et al., 2021), while tracking mobile prey or avoiding temporally variable predation risk (Kohl et al., 2018) may drive movement decisions over the course of hours to days (Suraci, Frank, et al., 2019). Examining how selection changes across finer temporal grains may elucidate relationships and potential trade‐offs between drivers of fine‐scale movement and selection.

If selection behaviour changes across temporal grain, the resolutions at which researchers choose to sample animal movement (minutes to hours to days) may implicitly represent separate hypotheses about the scale at which habitat covariates are relevant to an animal and may obscure dynamics occurring at other grains (Wheatley & Johnson, 2009; Wiens, 1989). Historically, habitat selection studies have been conducted at the temporal resolution at which GPS data were collected (usually 1–12 hr between subsequent GPS locations; Bastille‐Rousseau et al., 2018), often without explicit consideration of the implications of that choice. Advances in GPS collar technology now allow researchers to observe animal movement at a much higher resolution (Cagnacci et al., 2010), and thus to examine the drivers of animal movement and habitat selection at different temporal grains, ranging from short‐term, fine‐scale steps to movements over longer periods of time. If movement and/or habitat selection behaviours change with temporal grain, analyses conducted at a single temporal resolution may overlook grain‐dependent patterns that can shed light on behavioural processes.

Large carnivore conservation in human‐dominated environments is increasingly recognized as important to global conservation efforts (Carter & Linnell, 2016), and understanding large carnivore spatial ecology in these systems can elucidate mechanisms that enable human–carnivore coexistence (Suraci, Frank, et al., 2019). Globally, large carnivores experience high rates of anthropogenic mortality (Ripple et al., 2014). As a result, large carnivores spatially avoid anthropogenic features (Abrahms et al., 2015; Wilmers et al., 2013) and exhibit temporal shifts in activity and habitat use to minimize the risk of encountering humans (Ordiz et al., 2011; Suraci, Frank, et al., 2019; Wilmers et al., 2021). In addition to avoiding anthropogenic risk, large carnivores must also balance the high energetic demands that come with carnivory, including substantial time spent in locomotion required to regularly hunt and kill large‐bodied prey (Gorman et al., 1998). In some cases, avoiding anthropogenic risk and balancing high energetic demands may be in conflict with each other, for instance if areas of high resource quality (e.g. higher prey density) are also riskier. In such cases, animals are expected to exhibit temporal partitioning to avoid these areas during risky times (e.g. during the day, when humans are most active) but relax their avoidance during times of lower risk (Kronfeld‐Schor & Dayan, 2003). Carnivores may also face trade‐offs between energetically efficient movement and risk avoidance if avoiding human features results in energetically suboptimal movement strategies (e.g. moving through more rugged terrain; Nickel et al., 2021). Both energetic constraints and fear responses to humans are widespread across large carnivore species in different environments, but how they inform movement and habitat selection over shorter temporal grains remains unknown.

Here, we investigate scale‐dependent drivers of short‐term movement and habitat selection for populations of two large carnivore species, African lions *Panthera leo* and pumas *Puma concolor*, living in two very different human‐dominated environments: the livestock–wildlife rangelands of Laikipia County, Kenya, and the urban‐adjacent Santa Cruz Mountains of California. Both species demonstrate strong behavioural responses to anthropogenic features. In the rangeland system of Laikipia, African lions alternatively exhibit spatial and temporal avoidance of livestock herding, the primary human use of the landscape, but may trade off safety with prey availability due to the overlap of high‐quality herding areas and high‐quality habitat for native large herbivores (Oriol‐Cotterill et al., 2015; Oriol‐Cotterill et al., 2015; Suraci, Frank, et al., 2019). In the rugged Santa Cruz Mountains, low‐level residential housing is the primary anthropogenic land use. Pumas exhibit strong fear responses to human presence and avoid housing (Smith et al., 2017; Wilmers et al., 2013).

We used integrated step selection analysis (iSSA; Avgar et al., 2016) to compare lion and puma habitat selection and movement across eight temporal grains, ranging from 5 min to 12 hr. In modelling movement and habitat selection jointly, this approach allowed us to ask how habitat features related to energetic expenditure and anthropogenic risk impacted both the selection and movement processes for lions and pumas. We also asked whether and how the influence of covariates on movement and habitat selection changed with temporal grain, which could shed light on strategies that these species used to balance multiple goals in human‐dominated landscapes. Applying this analysis to two species—lions and pumas—allowed us to explore and identify commonalities in how large carnivores manage trade‐offs between energetics and risk via habitat selection across multiple scales.

## 2 MATERIALS AND METHODS

### 2.1 Study systems and GPS collaring

Laikipia County is located in northern Kenya. Our 1,040‐km^2^ study area was comprised of six commercial ranches consisting of semi‐arid *Acacia* savanna and open grasslands. These properties are managed for conservation as well as livestock production and support abundant native large herbivore populations, and use traditional livestock practices in which livestock are moved into bomas, or temporary livestock corrals, at night, and are let out to graze under the supervision of herders during the day (O'Brien et al., 2018). Bomas are the centres of human activity on the landscape, and humans present substantial risk to lions, with human‐caused deaths accounting for 117 out of 133 mortalities for monitored lions between 1999 and 2016 (L. Frank, unpubl. data). Bomas likely also represent the areas of increased prey availability for African lions given that boma locations overlap with high‐quality forage for native large herbivores. For further description of the study area, refer the studies by Frank (2011) and Oriol‐Cotterill, Macdonald, et al. (2015). The Laikipia study system has an elevational range from 1,271 to 1,931 m and is largely flat with some escarpments. Slope ranges from 0° to 34° with a median slope of 1.16°.

The Santa Cruz Mountains are in the Central Coast region of California, and consist of a gradient of human residential development, including open space areas as well as exurban, suburban and urban areas across the 2,800 km^2^ study area. Habitat types include mixed redwood *Sequoia sempervirens* forests, mixed oak (*Quercus* sp.) forests and chaparral. Pumas in the Santa Cruz Mountains experience high rates of anthropogenic mortality, accounting for 17 of 32 deaths of collared adults and subadults between 2008 and 2020 (A.C. Nisi, unpubl. data). For further description, refer the study by Wilmers et al. (2013). The Santa Cruz Mountains are more topographically rugged than Laikipia, with an elevational range from 0 to 1,333 m, slope ranging between 0° and 48° and a median slope of 4.38°.

Lions and pumas were captured and fitted with GPS collars set to record a GPS location every 5 min (Vectronics Aerospace GPS Plus or Vertex; see Appendix S1 for description of animal capture). Data were collected from 14 African lions (nine females and five males) and 20 pumas (10 females and 10 males) from 23 September 2014 to 14 February 2016 and from 15 May 2015 to 9 October 2018 respectively. All African lions were adults, and 17 of 20 pumas were adults, with the remainder being subadults (ranging from 17 to 20 months).

### 2.2 Statistical analyses

We used iSSA to quantify habitat selection. Step selection analyses (SSAs) are a type of resource selection function (RSF; Johnson, 1980) that define availability based on movement, with available points generated by simulating random steps from the movement path (Fortin et al., 2005; Thurfjell et al., 2014). Integrated step selection analysis is a further extension of SSA, and allows for the movement and habitat selection processes, and how they are influenced by habitat covariates, to be modelled jointly (Avgar et al., 2016). The movement‐driven definition of availability makes temporal grain an important consideration for SSAs, since available points represent locations where an animal could have visited over a certain interval of time (Thurfjell et al., 2014). Applying iSSA to movement data at different temporal grains can thus allow us to compare drivers of movement and selection across temporal grains. We considered eight distinct temporal grains for this analysis: 5, 15 and 30 min, and 1, 2, 4, 8 and 12 hr. Because step lengths (distance between subsequent GPS locations) increase with temporal grain, the temporal resolution of GPS data used in habitat selection analyses is inherently linked to the spatial extent of analysis (Figures S1 and S2).

We subsampled 5‐min GPS tracks for lions and pumas to construct datasets at each temporal grain. For example, the 15‐min dataset was obtained by selecting every third 5‐min GPS location, and so on. Next, we excluded all non‐movement points, which for both species we defined as used points that were <20 m from the previous point for each dataset (Dickie et al., 2020). The 20 m cut‐off corresponds to the average GPS error for pumas in the Santa Cruz Mountains and agrees with empirically determined step length cut‐offs between stationary and moving behaviours for African lions in Laikipia (Suraci, Frank, et al., 2019).

For each dataset, we generated 20 available points for each used point by generating random step lengths and turning angles and projecting from the previous location. Step lengths were drawn from exponential (lions) and gamma (pumas) distributions fitted to the empirical data (Avgar et al., 2016). The choice of distribution was motivated by AIC and q–q plots. For both species, turning angles were drawn from Von Mises distributions fitted to the empirical data.

### 2.3 Habitat and movement covariates

For both African lions and pumas, models included anthropogenic features (distance to bomas for lions and housing density for pumas), topographic slope and per cent cover, all of which have been shown to be important for large carnivore movement (Nickel et al., 2021; Suraci, Frank, et al., 2019).

For African lions, boma locations were monitored for the duration of the study on the properties to which we had access. To account for the fact that African lions may have been responding to bomas on unmonitored neighbouring properties, locations that were <1 km from the study area boundary or >5 km away from the nearest active boma were excluded (as in Suraci, Frank, et al., 2019). Distance to boma was log‐transformed to account for the stronger response at short distances relative to longer distances (Suraci, Frank, et al., 2019).

For pumas, housing density was calculated by manually digitizing locations of houses from high‐resolution satellite imagery, and fitting an Epanechnikov kernel with a radius of 150 m around each housing point, which is the most informative spatial grain for puma movement (Wilmers et al., 2013). Housing density was cube‐root‐transformed to ameliorate its long right tail and make its coefficient more interpretable after covariate standardization.

Landscape topography is expected to strongly influence energetic expenditure during movement (Shepard et al., 2013); therefore, we included topographic slope for both species. We also included per cent vegetative cover (Appendix S2), which may provide hunting opportunities as well as offer more safety in areas close to people for both species. Topographic slope, per cent cover and housing density were rasterized at 30 m resolution.

To allow the joint inference on habitat selection and movement, we included movement covariates in all models: step length for lions and the natural log of step length for pumas, as recommended for step lengths drawn from exponential and gamma distributions respectively (Avgar et al., 2016). We also included directional persistence: cos(*θ_t_* − *θ_t_*_−1_), where *θ_t_* is the angle from the *x*‐axis of the step ending at the used or available point and *θ_t_*_−1_ is the angle of the prior step. Values range from −1 to 1, with values closer to 1 representing straighter movements.

All covariates were standardized (centred by mean and scaled by standard deviation) within each dataset to facilitate coefficient interpretation (Schielzeth, 2010). We used Pearson's correlation to test for collinearity between all pairs of covariates. No two pairs of covariates had an |*r*| > 0.17 for African lions or >0.36 for pumas.

### 2.4 Model fitting and interpretation

Coefficients were estimated via conditional logistic regression, fit with the *clogit* function from the survival package (Therneau, 2015). We used generalized estimating equations (GEE) to calculate robust standard errors to account for temporal autocorrelation (Appendix S3; Prima et al., 2017).

Model specification reflected a priori hypotheses about how carnivores balance avoiding risk from humans with energetic constraints and differed slightly between species according to study area characteristics. Model selection was conducted in three stages for each species. We predicted that both species may avoid human features more strongly during the day than at night, so we first tested for diel changes in the response to anthropogenic covariate by evaluating the support (quasi‐likelihood information criterion; QIC) of models that included (or not) a time‐of‐day interaction with the anthropogenic covariate alongside topographic slope, cover and movement covariates for each temporal grain. Models with ΔQIC < 2 were considered to have support (Pan, 2001). Next, we tested for interactions between habitat covariates by evaluating the respective support of candidate models differing in their covariate interaction structure. For both species, we predicted that proximity to human features may lead animals to relax their avoidance of slope in order to avoid risk from humans (Nickel et al., 2021), so we considered models that had an interaction between slope and bomas or housing density. We also hypothesized that lions may avoid bomas less strongly where there was higher vegetation cover that could allow them to move undetected (Suraci, Frank, et al., 2019); hence, we considered an interaction between boma and cover. Cover exhibited very low variation in the Santa Cruz Mountains (Figure S1), so we did not consider this interaction for pumas. For pumas, we also tested for a quadratic response to slope, hypothesizing that pumas may select for intermediate slopes that may allow them to reduce risk from people while avoiding high energetic costs of traversing very steep slopes. Because Laikipia is much flatter than the Santa Cruz Mountains, we did not include this interaction for lions. Finally, we considered whether habitat covariates mediated movement through models that included interactions between slope, cover and anthropogenic covariate with step length and directional persistence, hypothesizing that movement strategies may vary across risky to safe and rugged to flat areas. For example, animals may either speed up or slow down when near human risk to minimize exposure or increase crypsis (Suraci et al., 2019), and slower, more tortuous movement in rugged terrain likely would reflect how animals mediate movement behaviour to manage energetic constraints (Nickel et al., 2021). We selected a single model structure for each species to interpret across grains. When best‐supported model structure differed between grains, we chose the structure that received consistent support across grains (Appendix S4; Tables S1–S3). To interpret the effects of habitat covariates on selection across temporal grains, we calculated the relative selection strength (RSS) across the range of each focal habitat covariate relative to the same reference location across grains (Appendix S5; Avgar et al., 2017). To assess whether there were differences in habitat selection between males and females, we refit the top model separately for individuals from each sex. While all African lions were adults, there were three pumas that were <2 years old (all between 17 and 20 months) and that thus could be pre‐dispersal‐ or dispersal‐age (Logan & Sweanor, 2001). To ensure that puma results were not biased by age class, we refit the top model to data excluding the three individuals <2 years old and compared results with the model fit to all pumas.

While grain‐dependent responses may reflect meaningful behavioural processes (Wheatley & Johnson, 2009), it is also possible that grain‐dependent selection patterns could emerge purely as a function of changing availability domain (i.e. changes in the relative availability or distribution of different habitat types with changing temporal grain size) either through a functional response or patterns of spatial variation in covariates (Beyer et al., 2010). We compared covariate distributions (medians and upper and lower quartiles) at each temporal grain. If covariate availability was relatively constant across grains, a functional response was unlikely to have produced grain‐dependent patterns. To assess how patterns of covariate variation changed with temporal grain, we calculated the within‐strata variance (i.e. within groups of matched used and available locations), calculated as the mean variance of each matched‐case stratum, and the overall (across‐strata) variance at each temporal grain. Covariates that exhibited higher spatial autocorrelation would have particularly low within‐strata variance at short grains relative to long grains, and may thus impact fine‐grain selection less strongly than long‐grain selection.

We tested for spatial autocorrelation in model residuals using Moran's *I* correlograms (random subsets of 10,000 locations; 250 m increments; 1,000 bootstrapping iterations to estimate *p‐*values) as implemented in the ncf package (Bjornstad, 2020). Only one distance bin exhibited significant levels of spatial autocorrelation in residuals: 0–250 m for lions at the 8‐hr temporal grain (see Figure S3).

## 3 RESULTS

**FIGURE 1:**
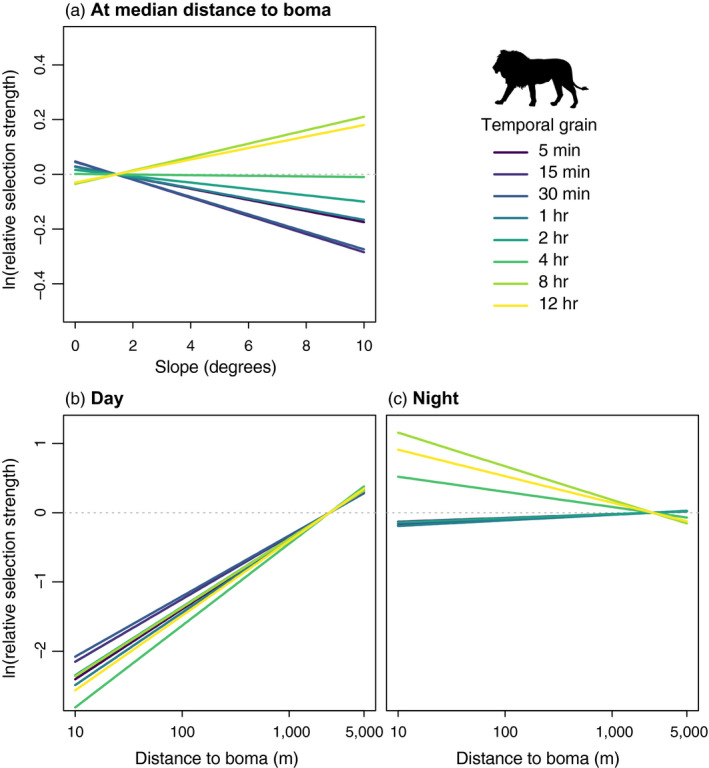
Relative selection strength of slope and distance to boma by lions across temporal grains. Selection strength was calculated relative to the same reference location across temporal grains, which had focal covariates (slope in panel a, distance to boma in panels b and c) set to their median values of 4‐hr available locations (Appendix S5). Non‐focal covariates (distance to boma in panel a, slope in panels b and c, and cover in all panels) were set to their median values of 4‐hr available locations, and movement covariates were set to their mean values for each temporal grain. Distance to boma is shown on the log scale

For African lions, topographic slope influenced habitat selection at shorter temporal grains but became unimportant at longer temporal grains (Figure 1a; Table 1). At the shortest grains (5–15 min), African lions exhibited significant avoidance of steeper slopes, but selection for slope was not significant at longer grains (≥1 hr). These results appear to be driven by females, with males exhibiting continued avoidance of steeper slopes at longer grains (Figure S4). Bomas influenced lion habitat selection differently between day and night. During the daytime, lions avoided locations closer to bomas across all temporal grains (Figure 1b). During the night‐time, lions relaxed this avoidance, and this relaxation was more pronounced for longer temporal grains, with lions switching to select locations closer to bomas at grains >4 hr (Figure 1c). Responses to bomas were similar between females and males (Figure S4).

**TABLE 1:** Coefficient estimates for lion iSSA. Robust standard errors are in parentheses and * and ** denote *p*‐values <0.05 and <0.01 respectively. ∆QIC values are shown relative to best‐supported model within each temporal grain (Table S2)

	5 min	15 min	30 min	1 hr	2 hr	4 hr	8 hr	12 hr
Boma	0.292** (0.099)	0.265** (0.069)	0.261** (0.061)	0.301** (0.057)	0.290** (0.054)	0.336** (0.060)	0.293** (0.059)	0.337** (0.075)
Boma*Night	−0.269** (0.105)	−0.229** (0.075)	−0.222** (0.067)	−0.261** (0.064)	−0.249** (0.063)	−0.379** (0.072)	−0.393** (0.085)	−0.393** (0.095)
Boma*Slope	0.032 (0.016)	0.059 (0.019)	0.078 (0.022)	0.065 (0.026)	0.086 (0.030)	0.056 (0.035)	0.094 (0.044)	0.161 (0.058)
Slope	−0.050** (0.015)	−0.086** (0.018)	−0.088** (0.021)	−0.057 (0.024)	−0.043 (0.028)	−0.012 (0.034)	0.047 (0.041)	0.023 (0.051)
Cover	−0.001 (0.009)	0.018 (0.011)	0.032 (0.014)	0.030 (0.017)	0.051 (0.021)	0.078 (0.026)	0.046 (0.033)	0.057 (0.040)
DP	0.125** (0.008)	0.068* (0.010)	0.030 (0.012)	0.011 (0.015)	−0.016 (0.018)	−0.021 (0.024)	0.034 (0.032)	0.035 (0.038)
SL	−0.117** (0.007)	−0.111** (0.011)	−0.078 (0.013)	−0.040 (0.016)	−0.006 (0.019)	−0.004 (0.025)	0.048 (0.031)	0.105 (0.035)
Boma*DP	−0.026* (0.007)	−0.020 (0.010)	−0.001 (0.012)	−0.012 (0.016)	0.011 (0.019)	0.046* (0.025)	−0.028 (0.034)	−0.054 (0.041)
Boma*SL	−0.029* (0.006)	−0.055** (0.009)	−0.074** (0.012)	−0.084** (0.014)	−0.104** (0.018)	−0.094** (0.023)	−0.070 (0.030)	−0.052 (0.036)
Slope*DP	−0.003 (0.006)	−0.001 (0.010)	−0.008 (0.012)	−0.025 (0.015)	0.029 (0.019)	−0.032 (0.023)	0.001 (0.029)	−0.032 (0.035)
Slope*SL	−0.165** (0.009)	−0.192** (0.014)	−0.216** (0.018)	−0.198** (0.022)	−0.196** (0.027)	−0.248** (0.035)	−0.115 (0.038)	−0.090 (0.042)
DP*SL	0.437** (0.010)	0.426** (0.013)	0.321** (0.014)	0.223** (0.016)	0.115** (0.018)	0.003 (0.022)	−0.072* (0.030)	−0.060 (0.034)
∆QIC	0.00	0.00	0.20	0.00	0.00	0.00	0.00	0.17

**FIGURE 2:**
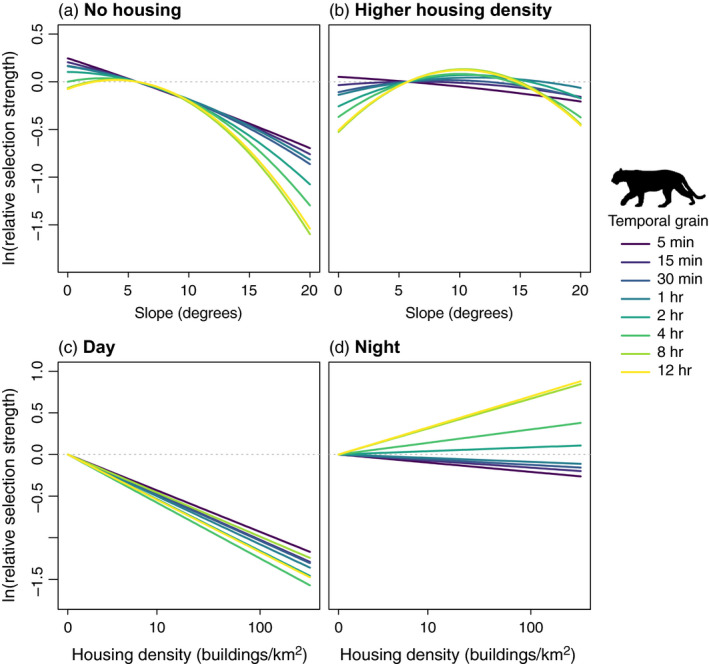
Relative selection strength of slope and housing density by pumas across temporal grains. Housing density was set at 0 and 28 houses/km^2^ in panels a and b respectively (lower and upper quartiles of 4‐hr available locations). Selection strength was calculated relative to the same reference location across temporal grains, which had focal covariates (slope in a and b, housing density in c and d) set to their median values of 4‐hr available locations (Appendix S5). Slope in panels c and d and cover in all panels were set to their median values of 4‐hr available locations, and movement covariates were set to their mean values for each temporal grain. Housing density is shown on the cube‐root scale

Pumas exhibited grain‐dependent selection for slope that was mediated by housing density (Figure 2, Table 2). For short‐grain movement (e.g. 5 min), pumas avoided steep slopes in areas without houses (0 houses/km^2^, Figure 2a), but relaxed this avoidance in areas of higher housing density (28 houses/km^2^, Figure 2b). For longer grain movement (e.g. 12 hr), pumas selected shallower slopes in areas with less development but selected intermediate slopes when housing density was higher. Males avoided steeper slopes more strongly than females, but grain‐dependent patterns of response to slope were similar between sexes (Figure S5). Similar to lions, pumas showed diel differences in their response to anthropogenic features. During the day, pumas avoided housing strongly across temporal grains (Figure 2c). At night, pumas relaxed their avoidance of housing and even exhibited selection for areas of higher housing density at longer temporal grains (Figure 2d). Responses to housing density were similar between females and males (Figure S6). Excluding the three pumas <2 years old did not influence our results (Figures S7 and S8).

**TABLE 2:** Coefficient estimates for puma iSSA. Robust standard errors are in parentheses and * and ** denote *p*‐values <0.05 and <0.01 respectively. ∆QIC values are shown relative to best‐supported model within each temporal grain (Table S3)

	5 min	15 min	30 min	1 hr	2 hr	4 hr	8 hr	12 hr
HD	−0.424** (0.022)	−0.481** (0.025)	−0.499** (0.028)	−0.534** (0.033)	−0.583** (0.036)	−0.657** (0.040)	−0.537** (0.043)	−0.675** (0.055)
HD*Night	0.339** (0.026)	0.420** (0.029)	0.453** (0.033)	0.505** (0.038)	0.646** (0.043)	0.834** (0.050)	0.927** (0.063)	1.088** (0.073)
HD*Slope	0.091** (0.004)	0.116** (0.006)	0.137** (0.008)	0.153** (0.011)	0.189** (0.014)	0.200** (0.019)	0.263** (0.025)	0.258** (0.029)
Slope	−0.106** (0.004)	−0.078* (0.007)	−0.056 (0.009)	−0.038 (0.012)	−0.009 (0.015)	0.031 (0.020)	0.082 (0.026)	0.096* (0.031)
Slope^2^	−0.004 (0.003)	−0.012 (0.004)	−0.022 (0.006)	−0.020 (0.008)	−0.041 (0.010)	−0.065* (0.014)	−0.090** (0.019)	−0.089* (0.022)
Cover	0.166** (0.005)	0.173** (0.008)	0.189** (0.010)	0.191** (0.013)	0.200** (0.016)	0.220** (0.021)	0.248** (0.028)	0.238** (0.032)
DP	0.039 (0.003)	0.064** (0.005)	0.062** (0.007)	0.053* (0.009)	0.026 (0.012)	0.005 (0.015)	0.005 (0.020)	0.005 (0.024)
ln(SL)	−0.100** (0.003)	−0.118* (0.005)	−0.105* (0.007)	−0.071 (0.009)	−0.014 (0.012)	−0.003 (0.015)	−0.010 (0.021)	0.005 (0.025)
HD*DP	−0.004 (0.003)	−0.009 (0.005)	−0.024 (0.007)	−0.027 (0.010)	−0.045** (0.012)	−0.045 (0.017)	−0.093** (0.023)	−0.101* (0.028)
HD*ln(SL)	0.010 (0.003)	0.014 (0.005)	0.017 (0.007)	0.008 (0.010)	−0.016 (0.013)	−0.063* (0.018)	−0.123** (0.025)	−0.150** (0.030)
Slope*DP	−0.053** (0.003)	−0.018 (0.006)	−0.020* (0.007)	0.013 (0.010)	0.003 (0.013)	0.026 (0.016)	−0.009 (0.022)	0.040 (0.027)
Slope*ln(SL)	−0.111** (0.003)	−0.120** (0.006)	−0.119** (0.008)	−0.123** (0.010)	−0.108** (0.013)	−0.085** (0.017)	−0.055 (0.023)	−0.056 (0.027)
Cover*DP	−0.095** (0.004)	−0.058** (0.006)	−0.029* (0.008)	−0.022* (0.010)	0.004 (0.013)	0.030 (0.017)	0.044 (0.023)	−0.012 (0.028)
Cover*ln(SL)	−0.003 (0.003)	−0.016 (0.006)	−0.022 (0.008)	−0.013 (0.010)	0.001 (0.013)	0.038 (0.018)	0.059* (0.024)	0.097** (0.028)
DP*ln(SL)	0.528** (0.003)	0.568** (0.005)	0.546** (0.007)	0.485** (0.009)	0.387** (0.011)	0.268** (0.014)	0.169** (0.019)	0.285** (0.023)
∆QIC	0.00	0.00	1.26	7.21	11.20	0.00	0.38	1.85

**FIGURE 3:**
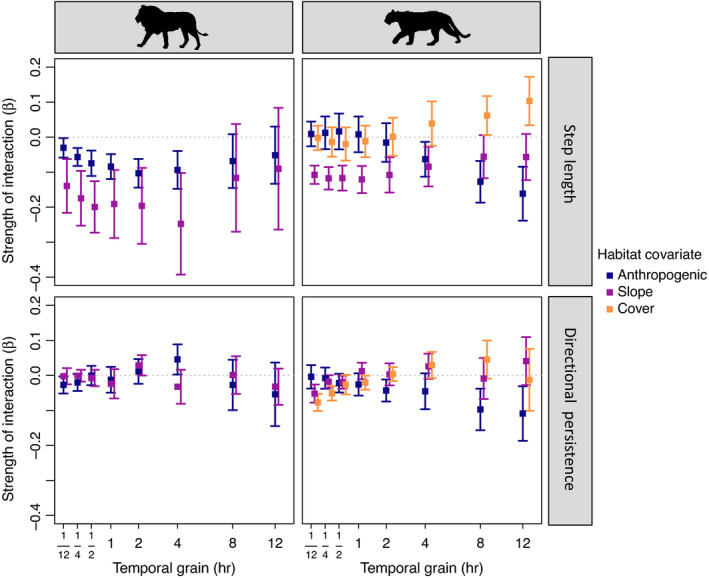
Effects of habitat covariates on lion and puma movement. For each species, the strength of interaction is the coefficient of the interaction between habitat and movement covariates multiplied by the same unit change in each habitat covariate (the standard deviation at the 4‐hr dataset for each species). Temporal grain is square‐root‐transformed for readability

Habitat covariates significantly influenced movement behaviour for both lions and pumas. Distance to boma and slope mediated lion movement (Figure 3; Table S2). At the 5‐min through 4‐hr temporal grains, there was a significant negative interaction between slope and step length, indicating that lions selected shorter steps and moved more slowly in areas with steeper slopes (Figure 3). Additionally, at the 5‐min through 4‐hr temporal grains, there were significant interactions between distance to boma and step length, with lions moving faster in areas closer to bomas. Habitat covariates did not strongly impact directional persistence for lions.

For pumas, there were significant interactions between step length and slope at short temporal grains (5 min through 2 hr), indicating that at these grains pumas moved slower where slope was steeper (Figure 3; Table S3). In contrast, housing density did not significantly influence puma movement at short grains, but did mediate step length at longer grains, where pumas selected shorter steps in areas of higher housing density. In addition, pumas selected more tortuous movement with increasing cover and slope at short temporal grains, and with increasing housing density at longer temporal grains, although the interactions between habitat covariates and directional persistence were of lower magnitude than for step length. Grain‐dependent patterns were largely consistent across sexes in how covariates influenced movement for both species (Figures S9 and S10).

The distributions of slope, cover and anthropogenic covariates in available locations were similar across grains (Figure S1). For each covariate, variance within matched‐case groups was less than overall variance and increased with temporal grain. This pattern was most pronounced for distance to boma (Figure S2).

## 4 DISCUSSION

Like most large carnivore species in human‐dominated environments, African lions and pumas must balance high energetic demands alongside the risk of anthropogenic mortality. Through a multi‐scale approach to habitat selection integrating both the movement and selection processes, we showed that the relative importance of these goals in driving large carnivore movement varied with temporal grain. While lions and pumas strongly avoided anthropogenic risk during the daytime at all temporal grains, landscape features related to energetic expenditure most strongly drove selection and movement over short temporal grains and tolerance of anthropogenic risk during the night‐time was more apparent at longer grains.

Both African lions and pumas avoided steeper slopes during movement at short temporal grains. Topographic slope is a strong determinant of energetic expenditure for large carnivores and most terrestrial species (Nickel et al., 2021; Shepard et al., 2013), and the strong influence of slope at fine temporal grains indicates that energetic constraints may be stronger drivers of short‐term rather than long‐term selection. Similarly, both lions and pumas took shorter steps, indicating slower movement, when slopes were steeper at short temporal grains, but slope did not mediate step length at longer grains. Animals may choose slower speeds to help mitigate the energetic costs of demanding terrain (Halsey, 2016; Shepard et al., 2013; Wilson et al., 2015), and here, slowing down when traversing steeper slopes likely reflects the trade‐off between energetic expenditure and time. Interestingly, the Laikipia region is much flatter overall compared to the Santa Cruz Mountains, so the fact that this fine‐scale avoidance of steep slopes was still seen across both systems suggests that locomotion‐driven energetic concerns are important drivers of carnivore movement even in flatter environments. For both lions and pumas, males avoided steeper slopes more strongly than females, and male lions exhibited avoidance of steeper slopes even across longer temporal grains. Males of both species engage in territorial patrol and generally range farther than females, which are associated with heightened importance of energetic constraints during locomotion (i.e. avoidance of steep slopes), and, therefore, likely drive the observed sex‐specific patterns (Benhamou et al., 2014; Johansson et al., 2018; Nickel et al., 2021).

Pumas also exhibited a grain‐dependent pattern in how they responded to slope in areas of higher risk from humans. Pumas avoided areas of steeper slopes across all temporal grains when housing density was low, but when risk from humans was higher, pumas did not respond to slope at fine grains and selected areas of intermediate slopes over longer term movement. These results indicate that carnivores prioritized avoiding risk from humans over energetic constraints when faced with a trade‐off between the two, consistent with previous findings (Nickel et al., 2021). Additionally, previous puma habitat selection studies have found both avoidance (Wilmers et al., 2013; Zeller et al., 2017) and selection of steeper slopes (Benson et al., 2016; Blecha et al., 2018). Our study demonstrates that puma response to slope is dependent both on temporal grain and exposure to risk from humans, so differential results may arise from the temporal grain of analysis and level of human presence on the landscape.

Both African lions and pumas also demonstrated temporally sensitive risk avoidance, avoiding human features during the daytime and relaxing this avoidance at night. In human‐dominated systems, many species have been shown to temporally shift their activity patterns to minimize overlap with humans (Gaynor et al., 2018; Oriol‐Cotterill, Macdonald, et al., 2015; Suraci, Frank, et al., 2019). In Laikipia and in the Santa Cruz Mountains, human activity around anthropogenic features is highest during the daytime, so daytime avoidance of these features likely functions to minimize the risk of encountering people. For both species, daytime avoidance of areas with more human influence was strong across temporal grains, underscoring that avoiding anthropogenic risk is crucial to large carnivores traversing human‐dominated landscapes. Additionally, this suggests that patterns of daytime avoidance over longer periods of time resulted from the scaling‐up of finer grained responses to anthropogenic features (Boyce et al., 2017; Prokopenko et al., 2017). Notably, this pattern holds for lion responses to bomas as well as puma responses to housing, despite the fact that bomas and houses are distributed markedly differently—bomas are rare landscape features and overall boma density is very low in Laikipia, while housing development covers a wide range of densities across the Santa Cruz Mountains. Large carnivore avoidance of risk from humans may lead to broader ecological effects through changes in carnivore impacts on prey behaviour, affecting space use by species across multiple trophic levels (Suraci, Clinchy, et al., 2019) with potential effects on primary producers (Yovovich et al., 2021).

While daytime avoidance was strong across temporal grains, the relaxation of this avoidance during the night‐time was stronger at longer temporal grains for both felids. When risky areas overlap with resource availability, animals are expected to use these areas for foraging during times of lower risk (Kronfeld‐Schor & Dayan, 2003). In Laikipia, bomas are located in areas of high‐quality livestock forage and thus likely overlapped substantially with habitat preferred by native large ungulates, and additionally may present a source of food themselves, in the form of domestic livestock—though previous studies have shown that wild prey account for the majority of lion kills even close to bomas (Suraci, Frank, et al., 2019). A previous study demonstrated that African lion selection for habitat near bomas was driven by feeding behaviour, suggesting that African lions balance costs and benefits from these risky but high‐value areas by using them during less risky times (Suraci, Frank, et al., 2019). Thus, night‐time selection for bomas may have been driven by hunting opportunities and resource acquisition, and these goals may be stronger drivers of long‐term rather than short‐term movements.

Pumas also exhibited night‐time selection for housing density at longer temporal grains, but the mechanism is less clear. Deer detection on camera traps is higher in more human‐dominated areas, which could indicate higher deer abundance (Smith et al., 2016). This could result either from deer responses to human subsidies (e.g. landscaping and lawn irrigation) or if deer were using more developed areas as ‘human shields’ (Berger, 2007; Hebblewhite et al., 2005). However, other studies have shown that pumas avoided housing density when killing deer and deer kill sites were disproportionately located in wildlands relative to more developed areas (Nickel et al., 2021; Wilmers et al., 2013), which is contrary to what would be expected if night‐time selection for housing density was driven by puma hunting deer in those areas. Several smaller mesocarnivore prey species are almost certainly more abundant nearer to people, but make up a much smaller percentage of puma diets (Smith et al., 2016). Thus, while it is possible that prey availability may drive night‐time selection, more investigation is needed to resolve this issue. Alternatively, it is possible that night‐time selection for housing density was in part a function of the strong avoidance that pumas exhibited during the day. In this fragmented landscape, pumas may need to move through more developed areas as they traverse their home ranges and choose to do this during relatively safer night‐time hours to allow for stronger daytime avoidance.

Interestingly, both how anthropogenic features mediated movement as well as the grain‐dependent patterns of these responses differed between lions and pumas. At short temporal grains, lions moved faster in areas closer to bomas, while pumas moved more slowly in areas of higher housing density over longer temporal grains. These different strategies may be due to the relative abundance of these features on the landscape—since bomas are fairly rare, it is possible that the optimal choice is to quickly move past them when they are encountered (Dickie et al., 2020), whereas pumas must slow down and move more tortuously to navigate carefully around areas of higher housing density that cover wide swaths of the Santa Cruz study area. A meta‐analysis synthesizing human impacts on animal movement also documented mixed responses (Doherty et al., 2021), and future work looking at movement responses to anthropogenic features for other large carnivores in human‐dominated landscapes could elucidate whether variations in speed responses are related to the density or distribution of risky features on the landscape. Additionally, the role of sociality may influence carnivore movement strategies around risky features—solitary pumas may be able to effectively avoid detection around humans by moving slowly, but group living may preclude cryptic movement near human‐dominated areas for social species like African lions. In this case, moving quickly through such areas may be a more effective strategy for lions to minimize the risk of detection and encounter. How animal sociality influences movement is an emerging research topic (Westley et al., 2018) and investigating these responses across other solitary and social carnivores may elucidate whether there are consistent patterns in how sociality mediates movement strategies around risky features. However, one commonality between species is that habitat covariates modified step length more strongly than directional persistence, indicating that these species modify their speeds to a greater degree than their tortuosity in relation to energetics and risk avoidance. Increased tortuosity in movement can present substantial energetic costs (Wilson et al., 2013), so an interesting future direction would be to explore the energetic costs of modifying speed versus tortuosity in response to risky features. While some broad‐scale movement patterns have been identified across taxa, including generally reduced movement in more human‐dominated areas and increased nocturnality (Gaynor et al., 2018; Tucker et al., 2018), variability in these patterns is still apparent across species and systems (Doherty et al., 2021)—quantifying the mechanisms behind patterns in movement and selection responses across species will be an exciting area of future research.

Alongside behavioural mechanisms, changes in characteristics of the availability domain may produce scale‐dependent patterns in habitat selection (Beyer et al., 2010; Laforge et al., 2016). First, selection may be related to the mean availability of habitat features—known as a functional response (Mysterud & Ims, 1998)—which can arise via behavioural processes or through statistical or sampling artefacts (Beyer et al., 2010; Holbrook et al., 2019; Laforge et al., 2016). Given the distributions of covariate values were consistent across grains (Figure S1) and since we calculated selection strength relative to the same reference location across grains (Figures 1 and 2), these grain‐dependent patterns are unlikely to have emerged primarily from functional responses. Second, a covariate that varies over large distances may exhibit minimal variation at the matched case level over short temporal grains, hence having a reduced impact on selection at short relative to longer temporal grains. Distance to boma exhibited this pattern (Figure S2), and while lions responded more strongly to bomas at longer temporal grains at night, during the day they exhibited equivalently strong avoidance across temporal grains. Furthermore, all covariates exhibited increased within‐strata variation with increasing temporal grain to some degree (Figure S2), so if spatial autocorrelation in covariates was solely responsible for grain‐dependent habitat selection patterns, we would expect to see stronger responses to all covariates with increasing temporal grain. Since this is not what we observed, we do not believe that this mechanism alone drove our results.

These two potential explanations for grain‐dependent patterns—scale‐dependent behavioural strategies and patterns of covariate variation—are not mutually exclusive, and both likely influence how habitat selection and movement change with the temporal grain of analysis. In our case, examining selection and movement across grains (a) revealed dynamics that would not have been apparent had only one temporal grain been considered (e.g. avoidance of steep slopes at fine grains and night‐time selection for anthropogenic features at longer temporal grains), and (b) suggested changes in the shape of the responses with temporal grain (e.g. slope for pumas). Selecting a single temporal grain (e.g. 5 min or 4 hr), as is typically done in habitat selection studies, would have resulted in qualitatively different conclusions about how lions and pumas responded to these features.

By considering how habitat covariates impacted movement and selection across temporal grains, our study sheds light on how large carnivores balance multiple, sometimes conflicting goals when traversing human‐dominated landscapes, providing novel insight on large carnivore behavioural ecology. Temporal grain is an important consideration in habitat selection studies, with energetic constraints driving carnivore movement over the short term, while diel partitioning of risk and resource acquisition influenced selection more strongly at longer temporal grains. Our results also suggest that avoiding anthropogenic risk may supersede energetic concerns for large carnivores in human‐dominated landscapes. Since results were consistent across two very different human‐dominated landscapes (a pastoral rangeland system and a fragmented, urban‐adjacent system) and for two very different species (the large, social African lion and smaller, solitary puma), these patterns may represent commonalities in large carnivore movement ecology across a variety of risky areas and landscapes of anthropogenic fear.

## CONFLICT OF INTEREST

The authors declare no conflict of interest.

## AUTHORS' CONTRIBUTIONS

A.C.N., J.P.S. and C.C.W. conceived the project idea and plan of analysis; S.E., L.G.F., A.O.‐C., C.C.W., T.M.W. and A.C.N. conducted fieldwork and collected the data; A.C.N. conducted analysis and led the writing of the manuscript with substantial input from J.P.S., N.R. and C.C.W. All authors gave meaningful contributions to this manuscript.

## Supporting information

Supplementary Material

## Data Availability

Data are available from the Dryad Digital Repository at https://doi.org/10.7291/D1J090 (Nisi et al., 2021).
