# Supplementary material for: Temporal scale of habitat selection for large carnivores: Balancing energetics, risk and finding prey
Source: J Anim Ecol. 2021 Oct 29;91(1):182–95. doi: 10.1111/1365-2656.13613 (PMC9298125; doi:10.1111/1365-2656.13613)
Supplement: Supplementary file 1 — Supplementary Material [file JANE-91-182-s001.docx]

**Title**

Temporal scale of habitat selection for large carnivores: balancing energetics, risk and finding prey

**Authors**

Anna C. Nisi, Justin P. Suraci, Nathan Ranc, Laurence G. Frank, Alayne Oriol-Cotterill, Steven Ekwanga, Terrie M. Williams, Christopher C. Wilmers

**Supporting Information**

**Appendix S1** – Animal capture and collaring

African lions and pumas were captured and collared under Animal Use Protocols No. 191 from UC Berkeley (issued to L. G. Frank) and WilmC1402 from UC Santa Cruz (issued to C. C. Wilmers). African lions were captured by using baits and audio calls, and free darting from a vehicle. Ketamine and medetomidine were used to anesthetize lions and were reversed with atipamezole ~1 hour after darting. Pumas were captured using trained hounds or box traps and were anaesthetized with Telazol. Both African lions and pumas were fitted with GPS collars set to record a GPS location every 5 minutes (Vectronics Aerospace GPS Plus or Vertex, Berlin, Germany). High-resolution (5-minute) spatial data were collected for an average of 55 days (range: 5-135) for 14 African lions and 60.1 days (range: 13-114) for 20 pumas.

**Appendix S2** – Calculating percent cover

Vegetative cover has been identified as an important landscape feature for movement and hunting behaviors for several large carnivore species (Boydston, Kapheim, Watts, Szykman, & Holekamp, 2003; Ordiz, Støen, Delibes, & Swenson, 2011), so we included percent vegetative cover as predictors for both African lion and puma analyses. We calculated percent cover for both species by first assigning cover or no cover indicators to vegetative data from both study systems. For Laikipia, we used the habitat layer from the Centre for Training and Integrated Research in ASAL Development for Laikipia^[[1]](#footnote-1)^, and for the Santa Cruz Mountains, we used California GAP Data^[[2]](#footnote-2)^. For both data sources, we sorted habitat categories into two classes, cover and no cover, and then we conducted a focal analyses to calculate the percent cover was taken over a 90m moving window using the *raster* package (Hijmans, 2019).

For lions, cover classes (0 or 1) were assigned to raster cells based on the classifications of low, medium, and high concealment described in (Suraci et al., 2019). Medium and high concealment were considered cover (1), with low concealment considered no cover (0).

For pumas, cover classes (0 or 1) were assigned to raster grid cells with the following classifications considered “cover”: Forest and woodland systems (CN Level 1); Developed (CN Level 2, within Human Use Land); Chaparral, Deciduous dominated savanna and glade, and Conifer dominated savanna (CN Level 2, within Shrubland, steppe and savanna systems); all Floodplain and riparian (CN Level 2, within Riparian and wetland systems) except for Inter-Mountain Basins Greasewood Flat and North American Warm Desert Wash (CN Level 3); and Harvested Forest - Northwestern Conifer Regeneration (CN Level 3), Recently burned forest (CN Level 3), Introduced Upland Vegetation - Treed (CN Level 3), Introduced Riparian and Wetland Vegetation (CN Level 3).

**Appendix S3** – Generalized Estimating Equations

In step selection analyses, non-independence in the data can arise from the high temporal resolution of GPS data (i.e., temporal autocorrelation) and/or variation in habitat selection behavior across individuals (Prima et al. 2017). Generalized estimating equations (GEE) can account for both of these potential sources of non-independence through specifying clusters of data, in which data are potentially correlated within clusters but independent across clusters (Prima et al. 2017). Bias is minimized when there are ≥20 independent clusters (Prima et al. 2017). To account for variation between individuals, separate clusters can be created for each individual. However, if no substantial variation in behavior is observed across individuals, then data from the same individual can be separated into multiple clusters via destructive sampling, where some amount of data is removed between subsequent clusters from the same individual to ensure that those clusters are temporally independent. Pumas tend to exhibit individual variability in selection of housing density, so destructive sampling would have been inappropriate, and each puma was treated as a separate cluster (N = 20 clusters). For African lions, individual variability in response to bomas was minimal so for all but one individual we created two clusters of data separated by at least 3 days (N=27 clusters total), which was identified in a previous analysis to be the amount of time after which temporal autocorrelation is negligible (Fortin et al., 2005; Suraci et al., 2019).

**Appendix S4** – Model selection

For both African lions and pumas, models that contained day/night interaction with the anthropogenic covariate were best-supported across all temporal grains, with models without interactions scoring >2 ΔQIC. For lions, there was support for models containing interactions between slope and distance to boma (Table S1), and interactions between slope and distance to boma with movement terms (Table S2). While the interaction between distance to boma and slope received support across temporal grains (Table S1), this interaction was not significant at any grain (Table 1). For pumas, models containing a quadratic slope term and interactions between slope and housing density were best-supported, and interactions between slope, housing density, and cover with movement terms also received support across temporal grains (Table S3).

**Appendix S5** – Relative selection strength

Relative selection strength (RSS) was calculated to visualize how slope and anthropogenic covariates impacted habitat selection at each temporal grain. RSS is calculated as the ratio of the relative probability of use of point *x_2_* to the relative probability of use of point *x*_1_­: RSS(x_2_, x_1_) = w(x_2_)/w(x_1_) (Avgar, Lele, Keim, & Boyce, 2017). Each panel in Figures 1 and 2 show how the natural log of RSS varies across the range of a particular focal covariate for point x_2_ (e.g., slope for Fig. 1A). In each panel, non-focal habitat covariates for both x_2_ and x_1_ were held constant, generally at their median value (or upper and lower quartiles for housing density in Fig. 2A-B), measured for 4-hour available locations. The value of the focal covariate at location x_1_ was also set to its median for 4-hour available locations. The 4-hour dataset was chosen to select constant covariate values, as it was an intermediate grain considered and one frequently used in habitat selection analyses. Movement covariates were set at their means for each temporal grain.

Thus, the RSS can be interpreted as the relative selection for a location with a particular value of the focal covariate relative to a location with the median value of that covariate at the 4-grain, with all other covariates held constant and assuming mean movement behavior for that temporal grain. Since the reference location (x_1_) was identical across temporal grains, this allows for cross-grain comparisons of RSS curves.

***Supplementary Figures and Tables***

Figure S1. Distributions of habitat and movement covariates across temporal grains. Point estimate shows the median value, with bars denoting upper and lower quartiles. Temporal grain is square-root-transformed for readability.

Figure S2. Variance in habitat covariates within and across matched-case strata for all temporal grains. Temporal grain is square-root-transformed for readability.

Figure S3. Spline correlograms of Moran’s *I* across distance for model residuals. Points denote Moran’s *I* calculated in 250m bins. Black points denote *p* < 0.05 (i.e., significant spatial autocorrelation) and white points denote *p >* 0.05.

Figure S4. Sex-specific relative selection strength of slope and distance to boma for lions across temporal grains. Selection strength was calculated relative to the same reference location across temporal grains as in Figure 1.

Figure S5. Sex-specific relative selection strength of slope by pumas across temporal grains. Selection strength was calculated relative to the same reference location across temporal grains as in Figure 2.

Figure S6. Sex-specific relative selection strength of housing density by pumas across temporal grains. Selection strength was calculated relative to the same reference location across temporal grains as in Figure 2.

Figure S7. Relative selection strength of slope and housing density for 17 pumas >2 years old across temporal grains. Selection strength was calculated relative to the same reference location across temporal grains as in Fig. 2.

Figure S8. Effects of habitat covariates on puma movement for 17 pumas >2 years old. The strength of interaction is the coefficient of the interaction between habitat and movement covariates multiplied by the same unit change in each habitat covariate as in Figure 3.

Figure S9. Sex-specific effects of habitat covariates on lion movement. The strength of interaction is the coefficient of the interaction between habitat and movement covariates multiplied by the same unit change in each habitat covariate as in Figure 3.

Figure S10. Sex-specific effects of habitat covariates on puma movement. The strength of interaction is the coefficient of the interaction between habitat and movement covariates multiplied by the same unit change in each habitat covariate as in Figure 3.

Table S1. Model selection for models with interactions between boma and slope and boma and cover for lions, with no interactions between habitat and movement covariates. All models also contain an interaction between boma and night.

| Temporal grain | Model | ∆QIC |
| --- | --- | --- |
| 5 min | Boma * Slope | 0.00 |
| 15 min | Boma * Slope | 0.00 |
| 30 min | Boma * Slope | 0.00 |
| 1 hr | Boma * Slope | 0.00 |
|  | Boma * Slope + Boma * Cover | 0.42 |
| 2 hr | Boma * Slope | 0.00 |
|  | Boma * Slope + Boma * Cover | 0.86 |
| 4 hr | Boma * Slope | 0.31 |
|  | Boma * Slope + Boma * Cover | 0.00 |
| 8 hr | Boma * Slope | 0.00 |
| 12 hr | Boma * Slope | 0.00 |

Table S2. Model selection for models with interactions between habitat covariates (boma, slope, cover) and movement covariates (step length, directional persistence) for lions. Models also contain an interaction between boma and slope and boma and night.

| Temporal grain | Model | ∆QIC |
| --- | --- | --- |
| 5 min | (Boma + Slope)*movement | 0.00 |
| 15 min | (Boma + Slope)*movement | 0.00 |
| 30 min | (Boma + Slope)*movement | 0.20 |
|  | (Boma + Slope + Cover)*movement | 0.00 |
| 1 hr | (Boma + Slope)*movement | 0.00 |
| 2 hr | (Boma + Slope)*movement | 0.00 |
|  | (Boma + Slope + Cover)*movement | 1.73 |
| 4 hr | (Boma + Slope)*movement | 0.00 |
| 8 hr | (Boma + Slope)*movement | 0.00 |
| 12 hr | No movement interactions | 0.00 |
|  | (Boma + Slope)*movement | 0.17 |

Table S3. Model selection for models with interactions between habitat covariates (housing density, slope, cover) and movement covariates (log(step length), directional persistence) for pumas. Models also contain an interaction between housing density and slope and a quadratic slope term.

| Temporal grain | Model | ∆QIC |
| --- | --- | --- |
| 5 min | (HD + Slope + Cover)*movement | 0.00 |
| 15 min | (HD + Slope + Cover)*movement | 0.00 |
| 30 min | (HD + Slope)*movement | 0.00 |
|  | (HD + Slope + Cover)*movement | 1.26 |
| 1 hr | (HD + Slope)*movement | 0.00 |
| 2 hr | (HD + Slope)*movement | 0.00 |
| 4 hr | (HD + Slope)*movement | 1.38 |
|  | (HD + Slope + Cover)*movement | 0.00 |
| 8 hr | (HD + Cover)*movement | 0.00 |
|  | (HD + Slope + Cover)*movement | 0.38 |
| 12 hr | (HD + Cover)*movement | 0.00 |
|  | (HD + Slope + Cover)*movement | 1.85 |

References

Avgar, T., Lele, S. R., Keim, J. L., & Boyce, M. S. (2017). Relative Selection Strength: Quantifying effect size in habitat- and step-selection inference. *Ecology and Evolution*, *7*(14), 5322–5330. doi: 10.1002/ece3.3122

Boydston, E. E., Kapheim, K. M., Watts, H. E., Szykman, M., & Holekamp, K. E. (2003). Altered behaviour in spotted hyenas associated with increased human activity. *Animal Conservation*, *6*(3), 207–219. doi: 10.1017/S1367943003003263

Fortin, D., Beyer, H. L., Boyce, M. S., Smith, D. W., Duchesne, T., & Mao, J. S. (2005). Wolves influence elk movements: Behavior shapes a trophic cascade in Yellowstone National Park. *Ecology*, *86*(5), 1320–1330.

Hijmans, R. J. (2019). *raster: Geographic Data Analysis and Modeling.*

Ordiz, A., Støen, O. G., Delibes, M., & Swenson, J. E. (2011). Predators or prey? Spatio-temporal discrimination of human-derived risk by brown bears. *Oecologia*, *166*, 59–67. doi: 10.1007/s00442-011-1920-5

Suraci, J. P., Frank, L. G., Oriol-Cotterill, A., Ekwanga, S., Williams, T. M., & Wilmers, C. C. (2019). Behavior-specific habitat selection by African lions may promote their persistence in a human-dominated landscape. *Ecology*, *100*(4), e02644. doi: 10.1002/ecy.2644

1. www.cetrad.org [↑](#footnote-ref-1)
2. https://www.usgs.gov/core-science-systems/science-analytics-and-synthesis/gap [↑](#footnote-ref-2)
